# Chikungunya virus infection in human microglial C20 cells induces mitochondria-mediated apoptosis

**DOI:** 10.3389/fcimb.2024.1380736

**Published:** 2024-04-23

**Authors:** Narendra Kumar, Rashmi Santhoshkumar, Manjunatha M. Venkataswamy

**Affiliations:** ^1^ Department of Neurovirology, National Institute of Mental Health and Neurosciences (NIMHANS), Bengaluru, India; ^2^ Department of Neuropathology, Electron Microscopy, Common Research Facility, NIMHANS, Bengaluru, India

**Keywords:** chikungunya virus, human microglia, C20 cells, apoptosis, growth-kinetics, transmission electron microscopy

## Abstract

**Introduction:**

Chikungunya virus (CHIKV) infection is associated with acute clinical manifestations and chronic joint inflammation. CHIKV has emerged as a significant causative agent of central nervous system (CNS) complications, including encephalitis and related sequelae. Microglial cells, crucial for immune responses and tissue repair in the CNS, play a vital role in the host response to viral infections, with their activation potentially leading to either protection or pathology. In this study, the infection biology of CHIKV in the C20 human microglial cell line was investigated.

**Methods:**

The permissiveness of C20 cells to CHIKV infection was assessed, and viral replication kinetics were compared to Vero E6 cells. Cytopathic effects of CHIKV infection on C20 cells were examined, along with ultrastructural changes using transmission electron microscopy. Additionally, apoptosis induction, mitochondrial membrane potential, and alterations in cell surface marker expression were evaluated by flow cytometry.

**Results:**

CHIKV infection demonstrated permissiveness in C20 cells, similar to Vero cells, resulting in robust viral replication and cytopathic effects. Ultrastructural analysis revealed viral replication, mature virion formation, and distinctive cytoplasmic and nuclear changes in infected C20 cells. CHIKV infection induced significant apoptosis in C20 cells, accompanied by mitochondrial membrane depolarization and altered expression of cell surface markers such as CD11c, CD14, and HLA-DR. Notably, decreased CD14 expression was observed in CHIKV-infected C20 cells.

**Discussion:**

The study findings suggest that CHIKV infection induces apoptosis in C20 microglial cells via the mitochondrial pathway, with significant alterations in cell surface marker expression, particularly CD14 that is linked with apoptosis induction. These observations provide valuable insights into the role of human microglial cells in the host response to CHIKV infection and contribute to the knowledge on the neuropathogenesis of this virus.

## 1 Introduction

Chikungunya virus (CHIKV) infection, commonly associated with acute clinical manifestations such as fever, rash and polyarthralgia, is reported to persist for several weeks and cause incapacitating chronic joint inflammation (Goupil and Mores, 2016). The virus is known to replicate predominantly in fibroblasts, which accounts for its tropism for muscles, joints, and skin (Young et al., 2019). CHIKV infection is reported to cause various complications of the central nervous system (CNS) that include encephalitis, seizures, meningoencephalopathy, myelitis and choroiditis associated with neurological sequelae (Arpino et al., 2009; Economopoulou et al., 2009; Inglis et al., 2016; Vu and Jungkind, 2017). Neurological manifestations are reported to be common among severe complications of CHIKV infection (Barr et al., 2018; Mehta et al., 2018). CHIKV encephalitis is reported to be associated with long-term consequences such as cognition issues, depression, confusion and memory loss (van Aalst et al., 2017). The detection of CHIKV RNA and isolation of the virus from the cerebrospinal fluid of patients with encephalitis suggest that the virus is able to invade the CNS (Tandale et al., 2009).

Macrophages are established as the primary target cells for CHIKV infection where the virus persists long term (Abere et al., 2012). Microglial cells, the resident macrophages of the brain, have an essential role in immune responses and tissue repair in the CNS. These cells are involved in constant surveillance of the CNS microenvironment and are activated during invasion by pathogens (Yang et al., 2010; Lenz and Nelson, 2018; Norris and Kipnis, 2019). Activation of microglia during viral infections could lead to either protection against the invading virus or pathology (Manocha et al., 2014; Kumari et al., 2016; Torres-Ruesta et al., 2020). Activated microglia upregulate MHC class I/II proteins for efficient antigen presentation to T lymphocytes entering the brain during inflammatory conditions (Aloisi, 2001). CHIKV infection has been described previously in human neuroblastoma and glioblastoma cell lines (Dhanwani et al., 2012; Abraham et al., 2013). However, there is lack of substantial knowledge on the role of microglial cells in the pathogenesis of CHIKV encephalitis. With microglia being the first line of defense in the CNS, this knowledge is crucial to gain insights into the CNS complications caused by CHIKV.

A study on the proteomics of CHIKV-infected human microglial cell line CHME-5 reported that the virus blocked the expression of several proteins required for the anti-viral innate immune response (Abere et al., 2012). Murine infection with CHIKV is reported to activate microglia though the virus does not replicate in these cells (Das et al., 2015). An *in vitro* study on murine microglial cells has shown them to be permissive to CHIKV infection and to undergo apoptosis (Abere et al., 2012). We recently reported that the human microglial cell line CHME-3 is highly permissive to CHIKV infection (Qadri et al., 2022). Another human microglial cell line, C20, was recently immortalized from primary glia derived from fresh CNS tissue which has been used to understand neuropathogenesis of HIV-1 (Garcia-Mesa et al., 2017; Rai et al., 2020). These cells were reported to have microglia-like morphology and express microglial cell surface markers. They also demonstrate the migratory, phagocytic and inflammatory response similar to primary microglia (Garcia-Mesa et al., 2017). We were interested in deciphering the infection biology of CHIKV in C20 human microglial cells with respect to permissibility, viral replication, ultrastructural changes and cell death mechanisms.

In the present study we sought to investigate the infection biology of CHIKV in the C20 human microglial cell line. We assessed the susceptibility of C20 cells to CHIKV and growth kinetics of the virus during the early and late phases of infection. Further, the study examined the cell viability, ultrastructural changes, the cell death mechanism and alterations in the surface expression of myeloid cell markers in CHIKV-infected C20 microglial cells.

## 2 Material and methods:

### 2.1 Virus stock and cell culture

CHIKV strain DRDE-06 (GenBank accession number: EF210157.2) without A226V mutation in the E1 gene was used (Ghosh et al., 2017). The human microglial C20 cell line was obtained from Dr. Udaykumar Ranga, Jawaharlal Nehru Centre for Advanced Scientific Research, Bengaluru and was grown in Dulbecco’s Modified Eagle’s medium (DMEM; Gibco, USA), supplemented with 10% Fetal Bovine Serum (FBS; Gibco, USA), 100 U/ml Penicillin, 100 µg/ml Streptomycin (Gibco, USA) and 100 ug/ml Normocin (Invivogen, USA) (complete medium - C20). The virus was propagated in Vero-CCL81 cells grown in DMEM supplemented with 10% FBS, 100 U/ml penicillin and 100 µg/ml streptomycin (Gibco, USA) (complete medium - Vero). Vero E6 was grown in complete medium – Vero.

### 2.2 Growth kinetics

Vero E6 and C20 cells (50,000 cells/well) were seeded in 24-well plates, incubated overnight with 5% CO_2_, and infected with CHIKV at MOI 1. Mock infection with DMEM alone was performed in control wells. The virus was allowed to adhere for 1 hour at 37°C with 5% CO_2_. The supernatant was removed after adsorption, and the cells were washed once with DMEM and incubated at 37°C with 5% CO_2_ for 72 h in respective complete medium. The culture supernatant from CHIKV-infected and mock-infected wells was collected at 6, 12, 24, 36, 48, 60, and 72 hours post infection (hpi) and stored at -80°C for viral quantification by standard plaque assay. The cells were observed at different time points during incubation and images were captured using a digital SLR camera (Nikon D3100, Japan). The viral titer in the supernatant was expressed as plaque forming units (PFU) per ml at each timepoint. The experiments were performed in triplicates.

### 2.3 Cell viability assay

To quantify the viability of CHIKV-infected Vero E6 and C20 cells, the MTT (3-(4,5-dimethylthiazol-2-yl)-2,5-diphenyltetrazolium bromide) assay was used (Mosmann, 1983). Briefly, in a 96-well plate, cells (2 x 10^4^ cells/well) were seeded, incubated overnight, and infected with CHIKV at MOI 1. After 1 hour of virus adsorption at 37°C with 5% CO_2_, the supernatant was removed, cells were washed once with fresh DMEM and incubated with the respective complete medium. MTT (10 mg/ml) was added to the wells at 24, 48, and 72 hpi and incubated at 37°C with 5% CO_2_ for 90 min till the formazan crystals developed in the cells. The formazan was then solubilized in dimethyl sulfoxide (DMSO) by incubating at 37°C with 5% CO_2_ for 30 min, and absorbance was measured. The average optical density value of CHIKV-infected cells was normalized to the average optical density value of mock-infected cells and the percent viability was calculated. The experiments were performed in triplicates.

### 2.4 Transmission electron microscopy

C20 cells (0.8 × 10^6^) were seeded in 60 mm sterile petri dishes and incubated overnight for attachment at 37°C with 5% CO_2_ and then infected with CHIKV at MOI 1. The virus was allowed to adhere for 1 h at 37°C with 5% CO_2_. Following incubation, the unadsorbed virus was removed and cells were washed once with fresh DMEM, and the cells were supplemented with complete medium – C20. Buffered glutaraldehyde (2.5%) was used to fix the mock-infected and CHIKV-infected C20 cells at 24 and 48 hpi for 40 mins, postfixed with 1% buffered osmium tetroxide for 1 h at 4°C, dehydrated using graded series of distilled alcohol (70–100%) at 4°C. Propylene oxide was used for clearing. Finally, cell pellets were embedded in Araldite CY212 resin (TAAB, UK). Ultramicrotome (Leica UC6, Germany) was used to cut the ultrathin sections, and were collected on copper grids and contrasted using saturated methanolic uranyl acetate and 0.2% lead citrate. Stained grids were visualized under TEM (JEM-1400 Plus Jeol, Tokyo) and images (electron micrographs) were captured.

### 2.5 Apoptosis assay

C20 cells (0.4 × 10^6^) were seeded in T25 tissue culture flask and incubated overnight for attachment at 37°C with 5% CO_2_ and then infected with CHIKV at MOI 1. CHIKV was allowed to adhere for 1 h at 37°C with 5% CO_2_. Following incubation, the unadsorbed virus was removed and the cells were washed once with fresh DMEM, and the cells were supplemented with complete medium – C20. Upon 72 hpi, the cells were trypsinized, pelleted and washed with PBS. Cells were stained in 1X binding buffer using BD Pharmingen FITC Annexin V Apoptosis Detection Kit (Cat- 556547; BD Biosciences) as per manufacturer’s instructions and FACSVerse flow cytometer (BD Biosciences) was used to acquire the data followed by data analysis on FlowJo (BD Biosciences) software. The experiments were performed in triplicates.

### 2.6 Mitochondrial viability assessment

C20 cells (1 × 10^6^) were seeded in T25 tissue culture flask and incubated overnight at 37°C with 5% CO_2_ and then infected with CHIKV at MOI 1. CHIKV was allowed to adhere for 1 h at 37°C with 5% CO_2_. Following incubation, the unadsorbed virus was removed and the cells were washed once with fresh DMEM, and the cells were supplemented with complete medium – C20. Upon 72 hpi, the cells were trypsinized, pelleted and washed with PBS. Cells were stained in 1X assay buffer using JC-1 working solution as per manufacturer’s instructions from BD Pharmingen™ MitoScreen (JC-1) (Cat- 551302; BD Biosciences) and data was acquired on FACSVerse flow cytometer (BD Biosciences) followed by data analysis in FlowJo (BD Biosciences) software. The experiments were performed in triplicates.

### 2.7 Cell surface markers by flow cytometry

C20 cells (0.4 × 10^6^) were seeded in T25 tissue culture flask and incubated overnight at 37°C with 5% CO_2_ and then infected with CHIKV at MOI 1. CHIKV was allowed to adhere for 1 h at 37°C with 5% CO_2_. The unadsorbed virus was removed and the cells were washed once with fresh DMEM, and the cells were supplemented with complete medium – C20. Upon 24 hpi, the cells were trypsinized, pelleted and washed with PBS. Cells were stained in PBS using V500 Mouse Anti-Human HLA-DR (Cat-561224; BD Biosciences), V450 Mouse Anti-Human CD14 (Cat-560349; BD Biosciences) and PeCy7 Mouse Anti-Human CD11c (Cat-561356; BD Biosciences) antibodies for 20 minutes as per manufacturer’s instructions. FACSVerse flow cytometer (BD Biosciences) was used to acquire the data followed by data analysis on FlowJo (BD Biosciences) software. The experiments were performed in triplicates.

### 2.8 Statistical analysis

Mean ± standard error of mean has been used to represent the results. Either Analysis of variance (ANOVA) followed by *post hoc* analysis (Dunnett’s) for multiple comparisons or Two-Way ANOVA followed by Sidak’s multiple comparison test have been used to analyze the statistical significance and unpaired t test was performed to compare two groups. GraphPad Prism software (GraphPad Prism, Version 8.0.2) was used to perform the statistical tests. A p-value of < 0.05 was considered significant.

## 3 Results

### 3.1 Human microglial C20 cell line permits CHIKV infection and replication

**Figure 1:**
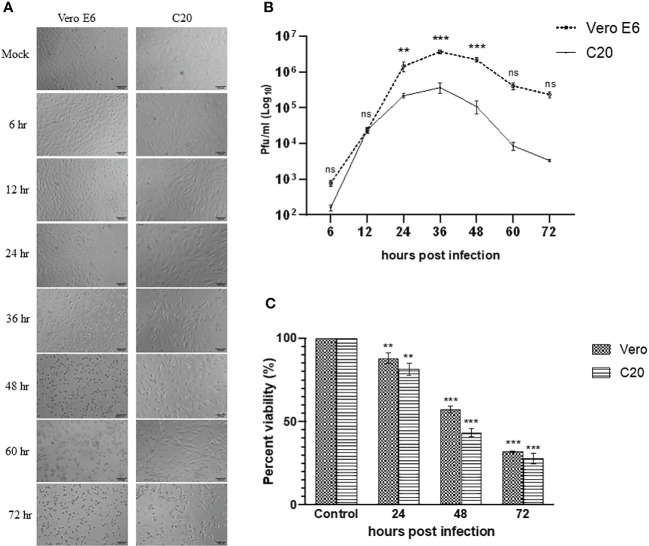
Infection of Vero E6 and C20 cells with Chikungunya virus at MOI 1. **(A)** Bright-field microscopy images of infected Vero E6 and C20 cells at different timepoints with both the cells showing cytopathic effect at later timepoints. Bar represents 100 µm. **(B)** Replication kinetics of CHIKV-infected Vero E6 and C20 cells. The values indicate the average number of plaques, with Vero E6 showing higher viral titer at most of the timepoints. Two way ANOVA followed by Sidak’s multiple comparison test have been used to analyze the statistical significance **(C)** Cell viability of mock and CHIKV-infected Vero E6 and C20 cells at different timepoints with decline in cell viability at later timepoints in both the cells. The data is presented as the mean ± standard error of mean. ANOVA followed by *post hoc* analysis (Dunnett’s) for multiple comparisons have been used to analyze the statistical significance. All the experiments were performed in triplicates. ** indicates p < 0.005, *** indicates p < 0.0001 and ns indicates non-significant.

The Vero E6 cells were highly susceptible to CHIKV infection and showed visible cytopathic effects (CPE) such as rounding, granulation, and detachment of cells as early as 24 hpi. The CHIKV-infected C20 cell line showed similar CPE starting 24 hpi and was very evident at 36 hpi. The images visualized by bright field microscopy at various time-points are depicted in **Figure 1A**.

The evaluation of viral replication kinetics at different time points in the C20 cell line revealed the presence of CHIKV as early as 6 hpi similar to the Vero E6 cell line with the highest viral titer at 36 hpi for both the C20 (~ 10^5^ pfu/ml) and Vero E6 (~ 10^6^ pfu/ml) cell lines (**Figure 1B**). The viral titer obtained with the C20 cell line was lower as compared to Vero E6, starting from 24 hpi until the late phase of infection.

### 3.2 Loss in viability of CHIKV-infected C20 cells

The MTT assay showed a decrease in viability of CHIKV-infected C20 and Vero E6 cells at 24 hpi. During the late phase at 48 and 72 hpi we observed a significant loss in viability among both the C20 and Vero E6 cells. Overall, the loss in viability among CHIKV-infected C20 cells was higher than the Vero E6 cells (**Figure 1C**).

### 3.3 Ultrastructural changes in CHIKV-infected C20 cells

**Figure 2:**
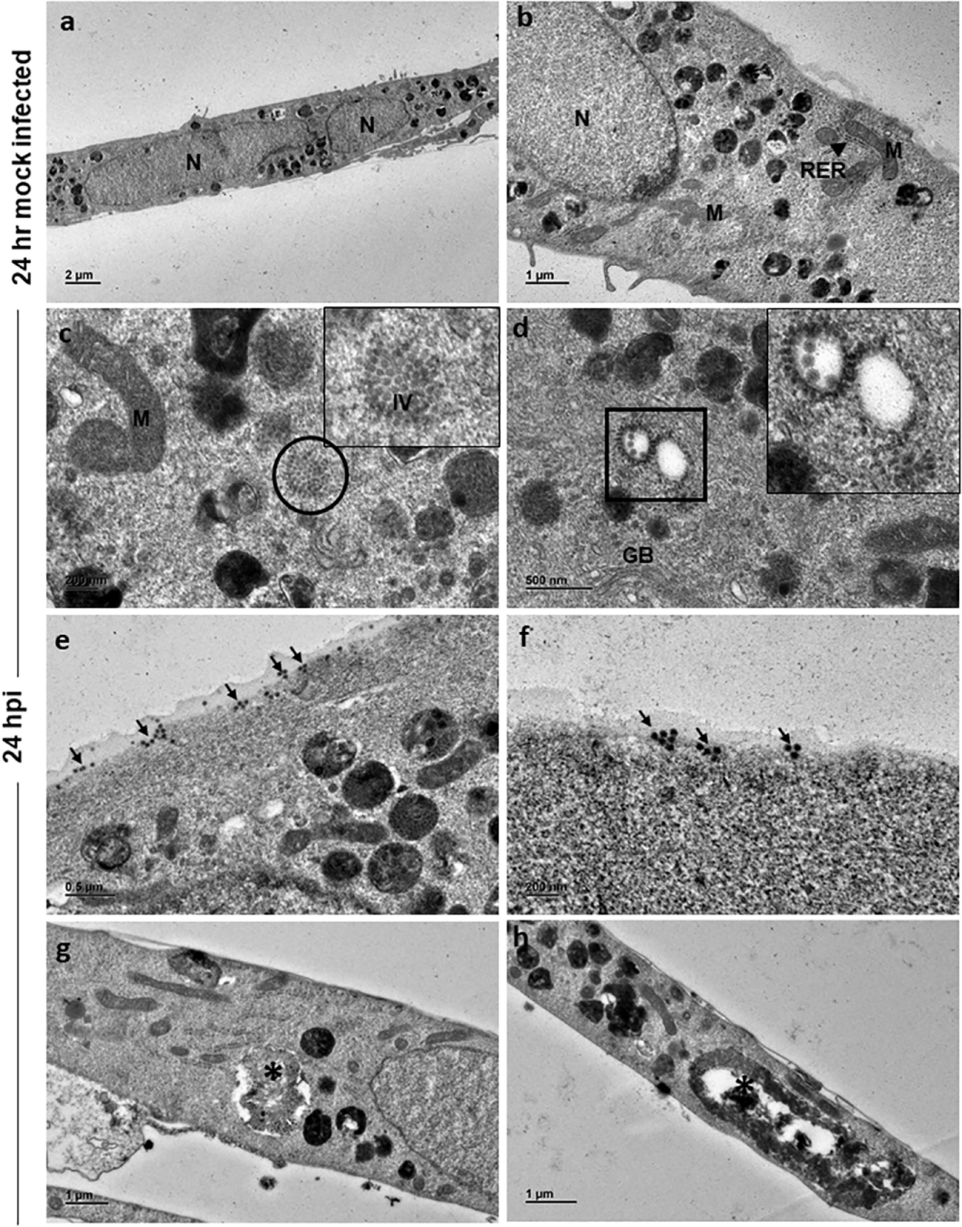
Electron micrographs of mock and CHIKV-infected C20 cells (MOI 1) at 24 hours post infection: **(A)** Low magnification micrograph of mock infected C20 cells showing normal contour, nuclei and numerous electron dense structures, x1000. **(B)** Normal sub-cellular structures like mitochondria (M), rough endoplasmic reticulum (RER, arrow), free ribosomes and numerous electron dense structures are seen at high magnification in mock infected C20 cells, x2500. **(C)** Numerous immature virions (IV) are present within the cytoplasm (circle) and abnormal mitochondria in CHIKV-infected C20 cells, x10000; Inset - high magnification showing collection of immature virions. **(D)** Orderly arrangement of numerous immature virions is seen on the membrane of cytoplasmic vesicle (square), golgi body (GB) is also seen, x10000. **(E)** Numerous viral particles (arrow) seen on both external and internal surface of the cell membrane, x6000. **(F)** Small clusters of mature viral particles (arrow) appear to be releasing from the cell membrane, x10000. **(G, H)** Membrane bound large cytopathic vacuoles (asterisk) with clear regions and with accumulation of electron dense degraded material with elongated mitochondria in CHIKV-infected C20 cells, x 3000.

Ultrastructural studies by TEM of 24 h and 48 h mock-infected and CHIKV-infected cells was performed to assess the structural features of normal C20 cells and alterations associated with CHIKV infection. The morphology of 24 h mock-infected cells appeared spindle shaped with long cellular processes. The nucleus was seen in the center of the cell (**Figure 2A**). Nucleoplasm consisted of uniform distribution of chromatin material and nucleus with electron dense nucleolus was noted occasionally. The cytoplasm contained various normal sub-cellular structures like Golgi apparatus, endoplasmic reticulum, mitochondria, polyribosomes and cytoskeletal filaments (**Figure 2B**). The cytoplasm was rich in membrane-bound structures containing pleomorphic electron dense material, and membranous whorled structures containing clear regions (**Figure 2B**).

CHIKV-infected C20 cells at 24 hpi retained the spindle shape and viral replication was apparent within the cell cytoplasm with numerous virions in clusters (**Figure 2C**, inset highlights the virions). Immature virions were seen with only an electron-dense core. Mature virions showed both an electron-dense core and an envelope. Clusters of immature virions within a membrane-bound vesicle and an orderly arrangement of virions on the external surface of the vesicle membrane facing the cytoplasmic region were appreciated (**Figure 2D**, inset highlights the virions). Mature virions were found singly and/or in clusters on both external and internal surface of the cell membrane (**Figure 2E**). Small groups of mature viruses were seen pinching off from the membrane (**Figure 2F**). The nuclei of infected cells displayed distinct euchromatin and electron dense heterochromatin condensed on the inner surface of the nuclear membrane. Cells with single membrane-bound large cytopathic vacuoles (CPV) containing degraded electron dense material and lipid droplets were observed. No abnormalities were noted in the subcellular structures (**Figures 2G, H**).

**Figure 3:**
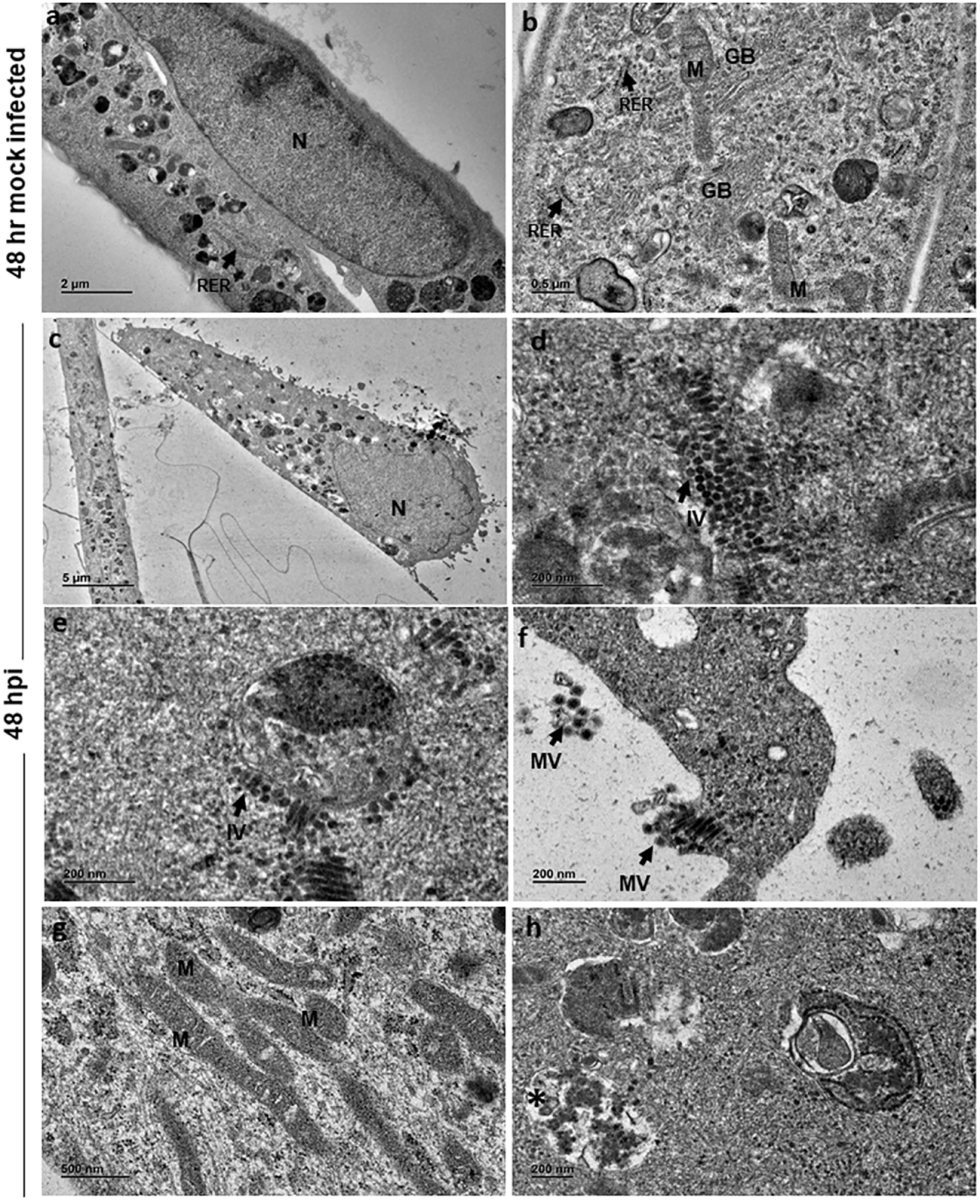
Electron micrographs of mock and CHIKV-infected C20 cells (MOI 1) at 48 hours post infection: **(A)** Low magnification micrograph of mock infected C20 cells showing normal contour, nucleus, numerous electron dense structures, x2000. **(B)** High magnification of mock infected C20 cells showing normal sub-cellular structures like mitochondria (M), rough endoplasmic reticulum (RER, arrow), free ribosomes, golgi body (GB) and numerous electron dense structures, x5000. **(C)** CHIKV-infected cell with eccentric nucleus (N) at low magnification, x300. **(D, E)** Numerous immature virions (IV, arrow) are seen in the cytoplasm freely and within membrane bound vesicle, x20000. **(F)** Clusters of mature virions (MV) released from infected cells, x15000. **(G)** Numerous slender and elongated mitochondria, x5000. **(H)** Membrane bound cytopathic vacuole (*) with clear regions and with accumulation of electron dense degraded material, x12000.

At 48 h, mock-infected cells showed features similar to that of 24 h (**Figures 3A, B**). However, at 48 hpi the cells appeared to be swollen with eccentrically placed nuclei containing distinct chromatin, nucleolus and dense cytoplasm (**Figure 3C**). Similar to 24 h infected cells, there were numerous immature virions seen in the cytoplasm (**Figure 3D**) in membrane bound vesicles and on the external surface of the vesicle (**Figure 3E**). Mature viral particles were seen in small clusters in close proximity to the cell boundaries (**Figure 3F**). Several slender and elongated mitochondria without alterations in cristae were seen (**Figure 3G**). Endoplasmic reticulum and Golgi bodies appeared normal. In addition, large cytopathic vacuoles (CPV) with single membrane-bound structure containing degraded electron dense material were observed (**Figure 3H**). An occasional cell with lipid droplets was also noted.

### 3.4 CHIKV infection induces apoptosis in C20 microglial cells

**Figure 4:**
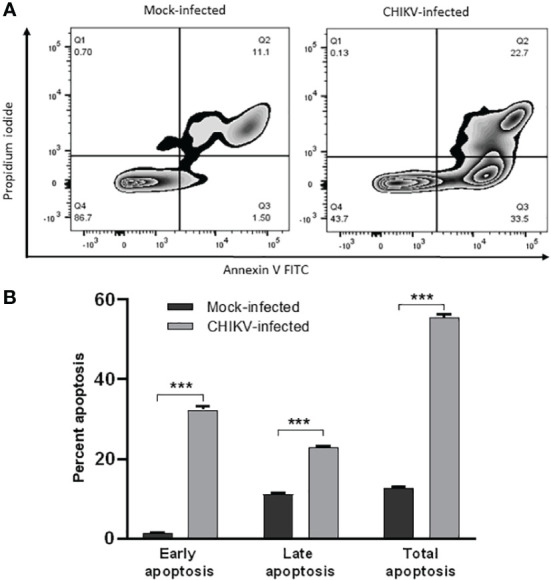
CHIKV infection induces apoptosis in C20 human microglial cells. **(A)** Zebra plot showing flow cytometric analysis of annexin-V and Propidium Iodide (PI) staining in mock-infected and CHIKV-infected C20 cells at 72 hours post infection **(B)** Early, late and total apoptosis of CHIKV-infected C20 cells at 72 hours post infection. The data is presented as the mean ± standard error of mean. Unpaired t test was performed to analyze statistical significance. The experiments were performed in triplicates. *** indicates p < 0.0001.

For apoptosis studies, annexin V positive cells were considered early apoptotic cells and, cells positive for both annexin V and propidium iodide (PI) were considered late apoptotic cells (**Figure 4A**). The mock-infected cells showed early apoptosis in 1.52% and late apoptosis in 11.3% at 72 hpi. CHIKV-infected cells showed early apoptosis in 32.4% and late apoptosis in 23.1% at 72 hpi. The total apoptosis observed in mock-infected cells was 12.82% as against 55.5% of CHIKV-infected cells (**Figure 4B**). Therefore, apoptosis following CHIKV infection in C20 microglial cells was significantly higher as compared to mock-infected cells.

### 3.5 CHIKV infection depolarizes mitochondrial membrane in C20 cells

**Figure 5:**
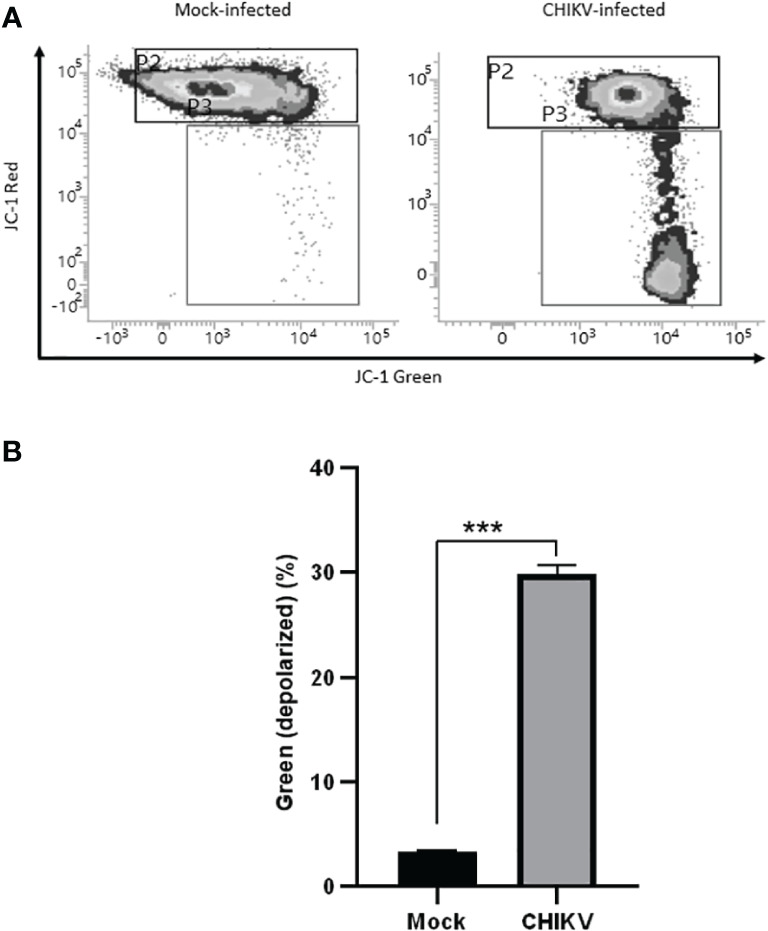
CHIKV infection causes mitochondrial membrane depolarization in C20 human microglial cells. **(A)** Density plot showing flow cytometric analysis of mitochondrial membrane depolarization by JC-1 dye staining in mock-infected and CHIKV-infected C20 cells at 72 hours post infection **(B)** Significant proportion of cells showing depolarization in CHIKV-infected C20 cells. The data is presented as the mean ± standard error of mean. Unpaired t test was performed to analyze statistical significance. The experiments were performed in triplicates. *** indicates p < 0.0001.

Mitochondrial membrane potential assessment was carried out at 72 hpi, JC-1 stained cells positive for red fluorescence were considered polarized while cells positive for green fluorescence were considered depolarized. The assay revealed depolarization in CHIKV-infected C20 cells at 72 hpi as shown in **Figures 5A, B**. About 30% of CHIKV-infected cells showed depolarization compared to 3% among mock-infected cells. For the data represented here, the fluorescence spill-over compensation was set to 70%. It is to be noted that despite lowering the compensation values, the proportion of polarized and depolarized cells did not exhibit significant changes, as depicted in **Supplementary Figure 1**. The utilization of a higher compensation value has been reported in a previous study (Perelman et al., 2012).

### 3.6 Alteration of cell surface markers in CHIKV-infected C20 cells

**Figure 6:**
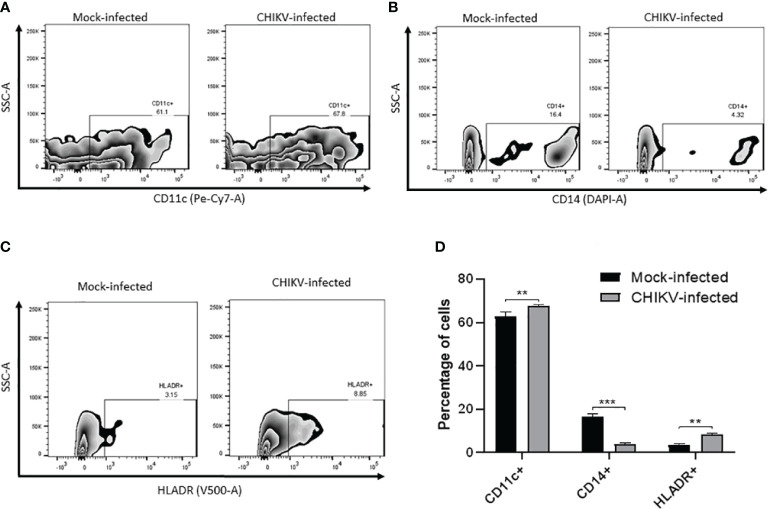
CHIKV infection causes alteration in cell surface markers in C20 human microglial cells. Zebra plot showing flow cytometric analysis of mock-infected and CHIKV-infected C20 human microglial cells to assess the surface expression of: **(A)** CD11c, **(B)** CD14, **(C)** HLA-DR and **(D)** percentage of cells showing either upregulation or downregulation of the markers. The data is presented as the mean ± standard error of mean. Unpaired t test was performed to analyze statistical significance. The experiments were performed in triplicates. ** indicates p < 0.05 and *** indicates p < 0.001.

We sought to evaluate the expression of certain cell surface expressed proteins typical of human myeloid derived microglia such as CD11c, CD14 and HLA-DR post CHIKV infection at 24 hpi. In the mock-infected C20 cells, CD11c was found to be expressed in 63% while CD14 was observed in 16.7% and HLA-DR was observed in 3.6% of the cells. In the CHIKV-infected C20 cells, CD11c was found to be expressed in 67.7% while CD14 was observed in 4% and HLA-DR was observed in 8.5% of the cells. The proportion of CHIKV-infected cells expressing CD11c and HLADR was significantly higher and those expressing CD14 were significantly lower than the mock infected cells (**Figure 6**).

## 4 Discussion

CHIKV is increasingly being reported as a cause of viral encephalitis, especially among children and elderly. It is therefore of high relevance to gain insights into the pathogenesis of CHIKV infection in microglial cells, the resident macrophages of the CNS. Microglia are important for the surveillance and maintenance of tissue homeostasis. They are the first ones to encounter an invading pathogen in the CNS and play a vital role in eliciting innate and adaptive immune responses to viral pathogens. In our previous study, the CHME3 human microglial cell line was found to be highly permissive to CHIKV infection (Qadri et al., 2022). The recently immortalized C20 human microglial cell line has been used to understand neuropathogenesis of Human Immunodeficiency Virus (HIV), integration of HIV-1 in microglial cells and neuroinflammation (Mazzeo et al., 2022; Rheinberger et al., 2023). Hence, we were interested in exploring the potential of CHIKV-infected C20 microglial cells as a model for studying neuropathogenesis of this virus.

The C20 microglial cell line was found to be highly permissive similar to the CHME3 microglial cell line. Further, these cells showed marked CPE and significant loss in viability right from the early phase of infection in contrast to the previous report on CHME3 cells, with no CPE or significant loss in viability (Qadri et al., 2022). These observations suggest that the C20 microglial cell line is highly susceptible to CHIKV infection and that microglia could serve as primary targets during CHIKV infection of the CNS.

Ultrastructural studies of CHIKV-infected C20 cells revealed swollen cells at 48 hpi with eccentrically placed nuclei, which are features of apoptotic cells (Sourisseau et al., 2007; Krejbich-Trotot et al., 2011; Hussain et al., 2016). Further, CPVs comparable to lysosomes and late endosomes, observed at 48 hpi are believed to be sites of replication for Alphaviruses (Froshauer et al., 1988; Kujala et al., 2001; Krejbich-Trotot et al., 2011; Gay et al., 2012; Hussain et al., 2016; Ghosh et al., 2018). The presence of such CPVs in infected cells may suggest an autophagic process. Replication of the virus was observed at both 24 and 48 hpi with virions in clusters and/or in orderly arrangements within the cells. The mitochondria appeared elongated at 48 hpi, which is suggestive of imbalance in mitochondrial dynamics in viral infection (Barbier et al., 2017). The budding of the viral particle was observed at 48 hpi, which is believed to be first mode of virus release (Chen et al., 2013; Hussain et al., 2016).

CHIKV-infected C20 cells showed significant apoptosis at 72 hpi, a major cell death mechanism reported in case of viral infections (Sourisseau et al., 2007; Krejbich-Trotot et al., 2011; Hussain et al., 2016). A previous study reported induction of apoptosis in human microglial cell line CHME-5 by the CHIKV isolates of the East, Central and South African (ECSA) Lineage (Wikan et al., 2012). The CHIKV-infected murine macrophage cell line, RAW264.7 is reported to undergo apoptosis through both intrinsic and extrinsic pathways (Nayak et al., 2017). Another study in CHIKV-infected HeLa cells showed that the virus hides in apoptotic blebs and infects neighboring cells by evading the host immune response (Krejbich-Trotot et al., 2011). Recently, the SARS-CoV-2 virus is shown to induce apoptosis of infected HMC3 human microglial cell line through both intrinsic and extrinsic pathways (Jeong et al., 2022).

To further investigate the cellular events that lead to apoptosis of CHIKV-infected C20 cells we probed the role of mitochondria by assessing the mitochondrial membrane potential. The interaction of viruses with mitochondrial membranes is known to result in depolarization of the mitochondrial membrane potential (MMP) and trigger the apoptotic cascade in infected cells (Foo et al., 2022). A notable fraction of CHIKV-infected C20 microglial cells exhibited depolarized MMP at 72 hours post-infection, suggesting mitochondrial impairment. Previous studies on other viral infections such as Dengue virus, SARS-CoV, and HIV too have demonstrated alteration of mitochondrial dynamics (Barbier et al., 2017; Tiku et al., 2020).

Apoptosis has been demonstrated to support viral replication with an increase in the number of infected cells. The human coronavirus, HCoV-OC43, has been reported to induce caspase dependent apoptosis in Vero cells and MRC-5 cells to promote viral replication (Maharjan et al., 2021). The increase in CHIKV titers observed in C20 microglial cells during late stage of infection may suggest a similar supportive role for apoptosis in viral replication. It is of note that a study on CHIKV-infected Mouse Embryonic Fibroblasts demonstrated the inhibitory role of autophagy against CHIKV-induced apoptosis resulting in reduced infection. The authors, however, suggest that autophagy is eventually overtaken by viral replication. The inhibition of autophagy resulted in increased apoptosis and higher proportion of infected cells (Joubert et al., 2012). In the present study, the presence of CPVs resembling autophagosomes in CHIKV-infected human microglial cells may suggest a similar cross-talk between autophagy and apoptosis.

The reduced surface expression of CD14 observed in CHIKV-infected C20 cells is likely to be associated with induction of apoptosis similar to previous studies on CHIKV in human monocytes and Rubella virus infection in human macrophages (Her et al., 2010; Schilling et al., 2022). Although we found a small subset of CHIKV-infected C20 microglial cells showing upregulation of HLA-DR, a previous study on CHIKV-infected human monocytes reported no alteration in the expression of this molecule (Her et al., 2010). Future studies are required to investigate whether the upregulation of HLA-DR in CHIKV-infected microglial cells is associated with antigen presentation and the innate immune response to CHIKV infection. Upon CHIKV infection, a small subset of C20 microglial cells showed upregulation of CD11c. Several studies have suggested a protective role for CD11c positive microglial cells in neuroinflammatory and neurodegenerative conditions (Wlodarczyk et al., 2014; Mayrhofer et al., 2021). Further research is required to understand the role of CD11c expression in microglial cells in the context of CNS viral infections.

Overall, the study findings provide valuable insights into the infection biology of CHIKV in C20 human microglial cells, highlighting the impact of the virus on cell viability, cellular ultrastructure and induction of the apoptotic process associated with mitochondrial dysfunction, and altered expression of surface myeloid cell proteins. The C20 microglial cell line offers another potential model for elucidating the interactions between CHIKV and microglial cells in the central nervous system. Understanding these mechanisms may aid in the development of targeted therapeutic strategies for CHIKV-associated neurological complications.

## 5 Conclusion

In conclusion, our study demonstrates that the C20 human microglial cell line is permissive to CHIKV infection. The infected cells exhibited cytopathic effects, including rounding, granulation, and detachment. CHIKV-infected C20 cells displayed a higher loss of viability compared to Vero E6 cells. Ultrastructural analysis of infected cells revealed presence of viral particles, altered nuclear morphology, and formation of cytopathic vacuoles resembling autophagosomes. Furthermore, CHIKV infection induced mitochondrial dysfunction and apoptosis in C20 cells. Additionally, CHIKV infection resulted in significant downregulation of surface CD14 expression, which is likely to be associated with apoptosis.

### 5.1 Limitations of the study

The study has not investigated whether the extrinsic apoptotic pathway is also involved in CHIKV-infected C20 microglial cell death. The assessment of variation in cell surface markers in CHIKV-infected C20 microglial cells is preliminary, the relevance of which needs to be examined thoroughly in future studies. The study has not probed whether CHIKV infection impacts autophagy in C20 microglial cells except for the findings from ultrastructural studies.

## Data availability statement

The original contributions presented in the study are included in the article/**Supplementary Material**. Further inquiries can be directed to the corresponding author.

## Author contributions

NK: Investigation, Formal analysis, Methodology, Visualization, Conceptualization, Writing – original draft, Writing – review & editing, Software, Validation. RS: Formal analysis, Methodology, Visualization, Writing – original draft. MV: Methodology, Visualization, Conceptualization, Funding acquisition, Project administration, Resources, Supervision, Writing – original draft, Writing – review & editing.

## References

[B1] Abere B. Wikan N. Ubol S. Auewarakul P. Paemanee A. Kittisenachai S.. (2012). Proteomic analysis of chikungunya virus infected microgial cells. PloS One 7, e34800. doi: 10.1371/journal.pone.0034800 22514668 PMC3326055

[B2] Abraham R. Mudaliar P. Padmanabhan A. Sreekumar E. (2013). Induction of cytopathogenicity in human glioblastoma cells by chikungunya virus. PloS One 8, e75854. doi: 10.1371/journal.pone.0075854 24086645 PMC3783433

[B3] Aloisi F. (2001). Immune function of microglia. Glia 36, 165–179. doi: 10.1002/glia.1106 11596125

[B4] Arpino C. Curatolo P. Rezza G. (2009). Chikungunya and the nervous system: what we do and do not know. Rev. Med. Virol. 19, 121–129. doi: 10.1002/rmv.606 19274635

[B5] Barbier V. Lang D. Valois S. Rothman A. L. Medin C. L. (2017). Dengue virus induces mitochondrial elongation through impairment of Drp1-triggered mitochondrial fission. Virology 500, 149–160. doi: 10.1016/j.virol.2016.10.022 27816895 PMC5131733

[B6] Barr K. L. Khan E. Farooqi J. Q. Imtiaz K. Prakoso D. Malik F.. (2018). Evidence of chikungunya virus disease in Pakistan since 2015 with patients demonstrating involvement of the central nervous system. Front. Public Health 6, 186. doi: 10.3389/fpubh.2018.00186 30042937 PMC6048291

[B7] Chen K. C. Kam Y. W. Lin R. P. T. Ng M. M. L. Ng L. F. Chu J. H. J. (2013). Comparative analysis of the genome sequences and replication profiles of chikungunya virus isolates within the East, Central and South African (ECSA) lineage. Virol. J. 10, 169. doi: 10.1186/1743-422X-10-169 23721429 PMC3679931

[B8] Das T. Hoarau J. J. Bandjee M. C. J. Maquart M. Gasque P. (2015). Multifaceted innate immune responses engaged by astrocytes, microglia and resident dendritic cells against Chikungunya neuroinfection. J. Gen. Virol. 96, 294–310. doi: 10.1099/vir.0.071175-0 25351727

[B9] Dhanwani R. Khan M. Bhaskar A. S. B. Singh R. Patro I. K. Rao P. V. L.. (2012). Characterization of Chikungunya virus infection in human neuroblastoma SH-SY5Y cells: role of apoptosis in neuronal cell death. Virus Res. 163, 563–572. doi: 10.1016/j.virusres.2011.12.009 22210004

[B10] Economopoulou A. Dominguez M. Helynck B. Sissoko D. Wichmann O. Quenel P.. (2009). Atypical Chikungunya virus infections: clinical manifestations, mortality and risk factors for severe disease during the 2005-2006 outbreak on Réunion. Epidemiol. Infect. 137, 534–541. doi: 10.1017/S0950268808001167 18694529

[B11] Foo J. Bellot G. Pervaiz S. Alonso S. (2022). Mitochondria-mediated oxidative stress during viral infection. Trends Microbiol. 30, 679–692. doi: 10.1016/j.tim.2021.12.011 35063304

[B12] Froshauer S. Kartenbeck J. Helenius A. (1988). Alphavirus RNA replicase is located on the cytoplasmic surface of endosomes and lysosomes. J. Cell Biol. 107, 2075–2086. doi: 10.1083/jcb.107.6.2075 2904446 PMC2115628

[B13] Garcia-Mesa Y. Jay T. R. Checkley M. A. Luttge B. Dobrowolski C. Valadkhan S.. (2017). Immortalization of primary microglia: a new platform to study HIV regulation in the central nervous system. J. Neurovirol. 23, 47–66. doi: 10.1007/s13365-016-0499-3 27873219 PMC5329090

[B14] Gay B. Bernard E. Solignat M. Chazal N. Devaux C. Briant L. (2012). pH-dependent entry of chikungunya virus into Aedes albopictus cells. Infect. Genet. Evol. J. Mol. Epidemiol. Evol. Genet. Infect. Dis. 12, 1275–1281. doi: 10.1016/j.meegid.2012.02.003 22386853

[B15] Ghosh A. Alladi P. A. Narayanappa G. Vasanthapuram R. Desai A. (2018). The time course analysis of morphological changes induced by Chikungunya virus replication in mammalian and mosquito cells. Acta Virol. 62, 360–373. doi: 10.4149/av_2018_403 30472865

[B16] Ghosh A. Desai A. Ravi V. Narayanappa G. Tyagi B. K. (2017). Chikungunya virus interacts with heat shock cognate 70 protein to facilitate its entry into mosquito cell line. Intervirology 60, 247–262. doi: 10.1159/000489308 29953983

[B17] Goupil B. A. Mores C. N. (2016). A review of Chikungunya virus-induced arthralgia: clinical manifestations, therapeutics, and pathogenesis. Open Rheumatol. J. 10, 129–140. doi: 10.2174/1874312901610010129 28077980 PMC5204064

[B18] Her Z. Malleret B. Chan M. Ong E. K. S. Wong S. C. Kwek D. J. C.. (2010). Active infection of human blood monocytes by Chikungunya virus triggers an innate immune response. J. Immunol. Baltim. Md 1950. 184, 5903–5913. doi: 10.4049/jimmunol.0904181 20404274

[B19] Hussain K. M. Lee R. C. H. Ng M. M. L. Chu J. J. H. (2016). Establishment of a novel primary human skeletal myoblast cellular model for chikungunya virus infection and pathogenesis. Sci. Rep. 6, 21406. doi: 10.1038/srep21406 26892458 PMC4759813

[B20] Inglis F. M. Lee K. M. Chiu K. B. Purcell O. M. Didier P. J. Russell-Lodrigues K.. (2016). Neuropathogenesis of chikungunya infection: astrogliosis and innate immune activation. J. Neurovirol. 22, 140–148. doi: 10.1007/s13365-015-0378-3 26419894 PMC4783292

[B21] Jeong G. U. Lyu J. Kim K. D. Chung Y. C. Yoon G. Y. Lee S.. (2022). SARS-coV-2 infection of microglia elicits proinflammatory activation and apoptotic cell death. Microbiol. Spectr. 10, e01091–e01022. doi: 10.1128/spectrum.01091-22 35510852 PMC9241873

[B22] Joubert P. E. Werneke S. W. de la Calle C. Guivel-Benhassine F. Giodini A. Peduto L.. (2012). Chikungunya virus-induced autophagy delays caspase-dependent cell death. J. Exp. Med. 209, 1029–1047. doi: 10.1084/jem.20110996 22508836 PMC3348111

[B23] Krejbich-Trotot P. Denizot M. Hoarau J. J. Jaffar-Bandjee M. C. Das T. Gasque P. (2011). Chikungunya virus mobilizes the apoptotic machinery to invade host cell defenses. FASEB J. Off Publ Fed Am. Soc. Exp. Biol. 25, 314–325. doi: 10.1096/fj.10-164178 20881210

[B24] Kujala P. Ikäheimonen A. Ehsani N. Vihinen H. Auvinen P. Kääriäinen L. (2001). Biogenesis of the semliki forest virus RNA replication complex. J. Virol. 75, 3873–3884. doi: 10.1128/JVI.75.8.3873-3884.2001 11264376 PMC114878

[B25] Kumari B. Jain P. Das S. Ghosal S. Hazra B. Trivedi A. C.. (2016). Dynamic changes in global microRNAome and transcriptome reveal complex miRNA-mRNA regulated host response to Japanese Encephalitis Virus in microglial cells. Sci. Rep. 6, 20263. doi: 10.1038/srep20263 26838068 PMC4738309

[B26] Lenz K. M. Nelson L. H. (2018). Microglia and beyond: innate immune cells as regulators of brain development and behavioral function. Front. Immunol. 9, 698. doi: 10.3389/fimmu.2018.00698 29706957 PMC5908908

[B27] Maharjan S. Kang M. Kim J. Kim D. Park S. Kim M.. (2021). Apoptosis enhances the replication of human coronavirus OC43. Viruses 13, 2199. doi: 10.3390/v13112199 34835005 PMC8619903

[B28] Manocha G. D. Mishra R. Sharma N. Kumawat K. L. Basu A. Singh S. K. (2014). Regulatory role of TRIM21 in the type-I interferon pathway in Japanese encephalitis virus-infected human microglial cells. J. Neuroinflammation 11, 24. doi: 10.1186/1742-2094-11-24 24485101 PMC3922089

[B29] Mayrhofer F. Dariychuk Z. Zhen A. Daugherty D. J. Bannerman P. Hanson A. M.. (2021). Reduction in CD11c+ microglia correlates with clinical progression in chronic experimental autoimmune demyelination. Neurobiol. Dis. 161, 105556. doi: 10.1016/j.nbd.2021.105556 34752925

[B30] Mazzeo A. Porta M. Beltramo E. (2022). Characterization of an immortalized human microglial cell line as a tool for the study of diabetic retinopathy. Int. J. Mol. Sci. 23, 5745. doi: 10.3390/ijms23105745 35628555 PMC9145666

[B31] Mehta R. Gerardin P. de Brito C. A. A. Soares C. N. Ferreira M. L. B. Solomon T. (2018). The neurological complications of chikungunya virus: A systematic review. Rev. Med. Virol. 28, e1978. doi: 10.1002/rmv.1978 29671914 PMC5969245

[B32] Mosmann T. (1983). Rapid colorimetric assay for cellular growth and survival: application to proliferation and cytotoxicity assays. J. Immunol. Methods 65, 55–63. doi: 10.1016/0022-1759(83)90303-4 6606682

[B33] Nayak T. K. Mamidi P. Kumar A. Singh L. P. K. Sahoo S. S. Chattopadhyay S.. (2017). Regulation of viral replication, apoptosis and pro-inflammatory responses by 17-AAG during chikungunya virus infection in macrophages. Viruses 9, 3. doi: 10.3390/v9010003 28067803 PMC5294972

[B34] Norris G. T. Kipnis J. (2019). Immune cells and CNS physiology: Microglia and beyond. J. Exp. Med. 216, 60–70. doi: 10.1084/jem.20180199 30504438 PMC6314530

[B35] Perelman A. Wachtel C. Cohen M. Haupt S. Shapiro H. Tzur A. (2012). JC-1: alternative excitation wavelengths facilitate mitochondrial membrane potential cytometry. Cell Death Dis. 3, e430–e430. doi: 10.1038/cddis.2012.171 23171850 PMC3542606

[B36] Qadri S. W. Kumar N. Santhoshkumar R. Desai A. Ravi V. Venkataswamy M. M. (2022) Infection of human microglial cell line CHME-3 to study neuropathogenesis of chikungunya virus. J. Neurovirol. 28(3):374–382. doi: 10.1007/s13365-022-01070-7 35352315

[B37] Rai M. A. Hammonds J. Pujato M. Mayhew C. Roskin K. Spearman P. (2020). Comparative analysis of human microglial models for studies of HIV replication and pathogenesis. Retrovirology 17, 35. doi: 10.1186/s12977-020-00544-y 33213476 PMC7678224

[B38] Rheinberger M. Costa A. L. Kampmann M. Glavas D. Shytaj I. L. Sreeram S.. (2023). Genomic profiling of HIV-1 integration in microglia cells links viral integration to the topologically associated domains. Cell Rep. 42, 112110. doi: 10.1016/j.celrep.2023.112110 36790927

[B39] Schilling E. Pfeiffer L. Hauschildt S. Koehl U. Claus C. (2022). CD14 is involved in the interferon response of human macrophages to rubella virus infection. Biomedicines 10, 266. doi: 10.3390/biomedicines10020266 35203475 PMC8869353

[B40] Sourisseau M. Schilte C. Casartelli N. Trouillet C. Guivel-Benhassine F. Rudnicka D.. (2007). Characterization of reemerging chikungunya virus. PloS Pathog. 3, e89. doi: 10.1371/journal.ppat.0030089 17604450 PMC1904475

[B41] Tandale B. V. Sathe P. S. Arankalle V. A. Wadia R. S. Kulkarni R. Shah S. V.. (2009). Systemic involvements and fatalities during Chikungunya epidemic in India, 2006. J. Clin. Virol. Off Publ Pan Am. Soc. Clin. Virol. 46, 145–149. doi: 10.1016/j.jcv.2009.06.027 19640780

[B42] Tiku V. Tan M. W. Dikic I. (2020). Mitochondrial functions in infection and immunity. Trends Cell Biol. 30, 263–275. doi: 10.1016/j.tcb.2020.01.006 32200805 PMC7126537

[B43] Torres-Ruesta A. Teo T. H. Chan Y. H. Rénia L. Ng L. F. P. (2020). Pathogenic Th1 responses in CHIKV-induced inflammation and their modulation upon Plasmodium parasites co-infection. Immunol. Rev. 294, 80–91. doi: 10.1111/imr.12825 31773780 PMC7064921

[B44] van Aalst M. Nelen C. M. Goorhuis A. Stijnis C. Grobusch M. P. (2017). Long-term sequelae of chikungunya virus disease: A systematic review. Travel Med. Infect. Dis. 15, 8–22. doi: 10.1016/j.tmaid.2017.01.004 28163198

[B45] Vu D. M. Jungkind D. (2017). Angelle desiree laBeaud. Chikungunya Virus Clin. Lab. Med. 37, 371–382. doi: 10.1016/j.cll.2017.01.008 28457355 PMC5469677

[B46] Wikan N. Sakoonwatanyoo P. Ubol S. Yoksan S. Smith D. R. (2012). Chikungunya virus infection of cell lines: analysis of the East, Central and South African lineage. PloS One 7, e31102. doi: 10.1371/journal.pone.0031102 22299053 PMC3267766

[B47] Wlodarczyk A. Løbner M. Cédile O. Owens T. (2014). Comparison of microglia and infiltrating CD11c^+^ cells as antigen presenting cells for T cell proliferation and cytokine response. J. Neuroinflammation 11, 57. doi: 10.1186/1742-2094-11-57 24666681 PMC3987647

[B48] Yang I. Han S. J. Kaur G. Crane C. Parsa A. T. (2010). The role of microglia in central nervous system immunity and glioma immunology. J. Clin. Neurosci. Off J. Neurosurg. Soc. Australas 17, 6–10. doi: 10.1016/j.jocn.2009.05.006 PMC3786731 19926287

[B49] Young A. R. Locke M. C. Cook L. E. Hiller B. E. Zhang R. Hedberg M. L.. (2019). Dermal and muscle fibroblasts and skeletal myofibers survive chikungunya virus infection and harbor persistent RNA. PloS Pathog. 15, e1007993. doi: 10.1371/journal.ppat.1007993 31465513 PMC6715174

