# Supplementary material for: Chikungunya virus infection in human microglial C20 cells induces mitochondria-mediated apoptosis
Source: Front Cell Infect Microbiol. 2024 Apr 23;14:1380736. doi: 10.3389/fcimb.2024.1380736 (PMC11074397; doi:10.3389/fcimb.2024.1380736)
Supplement: Supplementary file 1 [file DataSheet_1.pdf]

Chikungunya virus infection in human microglial C20 cells induces mitochondria-mediated apoptosis

Narendra Kumar[1], Rashmi Santhoshkumar[2], Manjunatha M. Venkataswamy[1#]

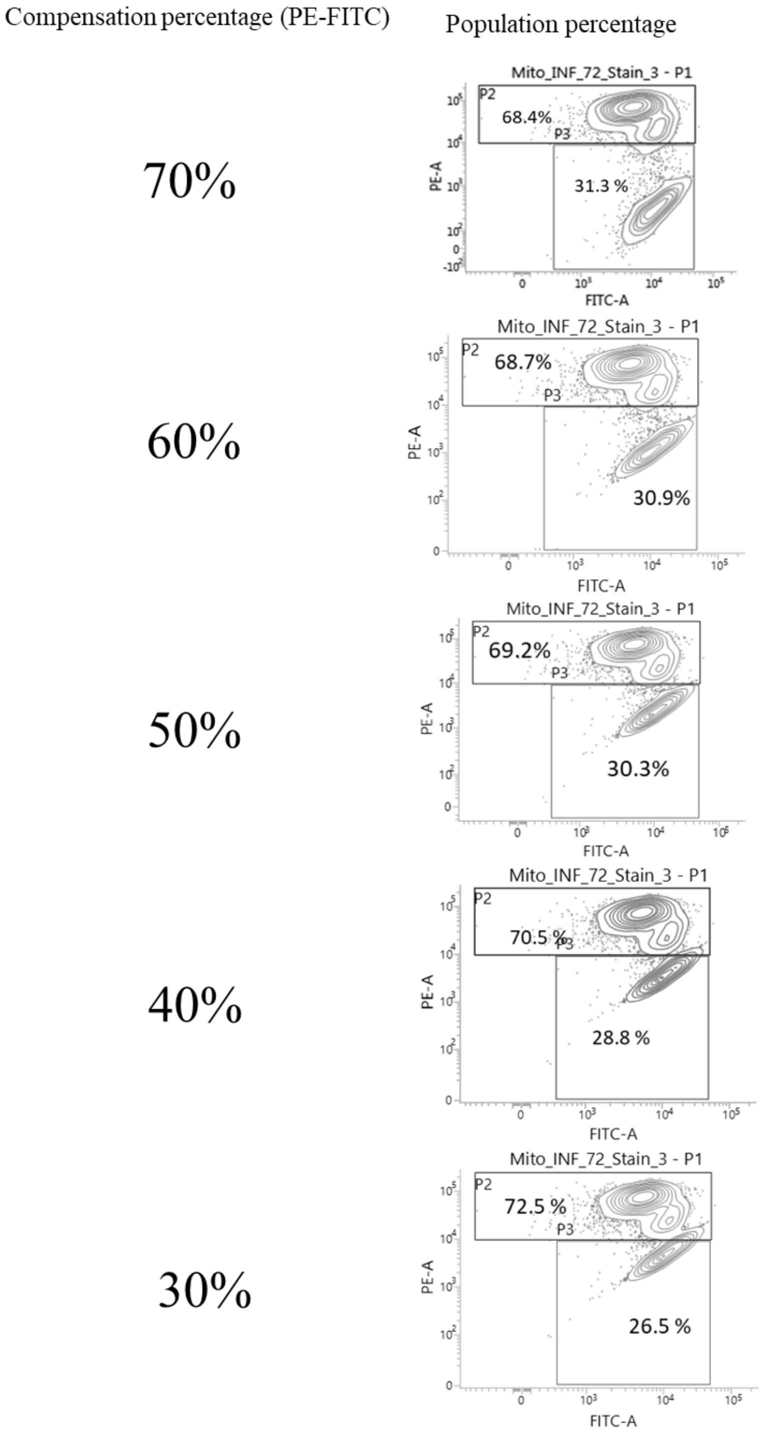

Supplementary Figure 1: Dots plots showing flow cytometric analysis by JC-1 dye staining of mock-infected and CHIKV-infected C20 human microglial cells. Similar proportions of cells (26.5% to 31.3%) with depolarized mitochondrial membrane potential were observed at different compensation values ranging from 30% at 70%.
